# Evolving Interplay Between Dietary Polyphenols and Gut Microbiota—An Emerging Importance in Healthcare

**DOI:** 10.3389/fnut.2021.634944

**Published:** 2021-05-24

**Authors:** Suman Kumar Ray, Sukhes Mukherjee

**Affiliations:** ^1^Independent Researcher, Bhopal, India; ^2^Department of Biochemistry, All India Institute of Medical Sciences, Bhopal, India

**Keywords:** dietary polyphenols, gut microbiota, gut health environment, bioavailability, bioactivity

## Abstract

Polyphenols are natural plant compounds and are the most abundant antioxidants in the human diet. As the gastrointestinal tract is the primary organ provided to diet sections, the diet may be regarded as one of the essential factors in the functionality, integrity, and composition of intestinal microbiota. In the gastrointestinal tract, many polyphenols remain unabsorbed and may accumulate in the large intestine, where the intestinal microbiota are most widely metabolized. When assuming primary roles for promoting host well-being, this intestinal health environment is presented to the effect of external influences, including dietary patterns. A few different methodologies have been developed to increase solvency and transport across the gastrointestinal tract and move it to targeted intestinal regions to resolve dietary polyphenols at the low bioavailability. Polyphenols form a fascinating community among the different nutritional substances, as some of them have been found to have critical biological activities that include antioxidant, antimicrobial, or anticarcinogenic activities. Besides, it affects metabolism and immunity of the intestines and has anti-inflammatory properties. The well-being status of subjects can also benefit from the development of bioactive polyphenol-determined metabolites, although the mechanisms have not been identified. Even though the incredible variety of health-advancing activities of dietary polyphenols has been widely studied, their effect on intestinal biology adaptation, and two-way relationship between polyphenols and microbiota is still poorly understood. We focused on results of polyphenols in diet with biological activities, gut ecology, and the influence of their proportional links on human well-being and disease in this study.

## Introduction

**Figure 1:**
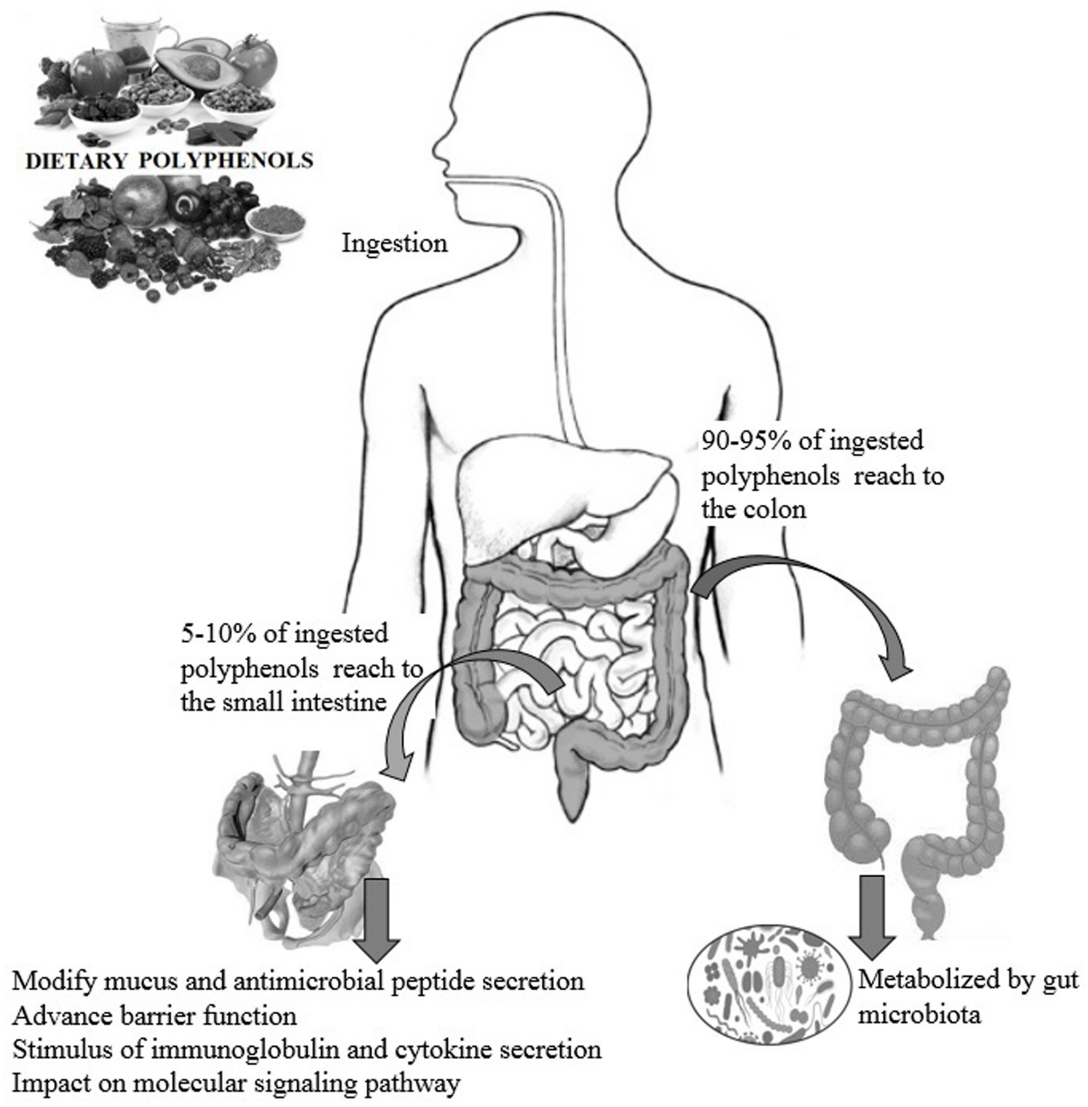
The impact of polyphenols on gut health and the possible modes of action (14).

Dietary polyphenols are natural plant-based compounds, including foods such as vegetables, cereals, fruits, coffee, tea, wine, etc. (1). Hydroxylated phenyl moieties define polyphenols as nothing but a large heterogeneous group of compounds. Polyphenols are usually categorized into flavonoids and non-flavonoids because of their chemical structure and complexity (including phenolic rings and substitution groups) (2). Human body perceives polyphenols as xenobiotics, and their bioavailability is moderately poor (3). Up until now, more than 8,000 polyphenols have been identified and are classified by their carbon skeleton concept: flavonoids, phenolic acids, lignans, and stilbenes (4). The intake of polyphenols varies by world topography, possibly because of the distinctive dietary habits of different cultures worldwide, as shown in numerous studies (5, 6). Micronutrient-rich essential diets with polyphenols are written in Tables 1, 2. It has been shown that phenolics and their metabolites have an affirmative influence on gut health by promoting the production of beneficial microbiota and controlling the propagation of pathogenic bacteria (8). In around 400 B.C., Hippocrates said, “death sits in the intestines” and “bad digestion is the root of all evil,” indicating the essential role of the human intestine in their health and disease. In most fruits and vegetables, polyphenols, along with herbs and spices, are secondary plant metabolites (9, 10). In particular, due to structural complexity, polyphenols with higher molecular weight avoids absorption in the small intestine. It has been shown that in the small intestine, simply 5–10% of the overall ingested polyphenols may be absorbed (11), and how the health-advancing effects of polyphenols arise when they are inadequately ingested and commonly identified at trace levels in the systemic circulation should be considered. A complex microbial ecosystem is present in the human digestive tract, including extensive metabolic versatility, using metabolic pathways that people have not created (12). As a result, it was calculated that the advanced well-being effects of phenolics, for example, polyphenols, may possibly be detected by regulation of the composition and act of the gut microbiota, or vice versa, by the generation of bioactive microbial metabolites. Like this, the colonic microbiota plays an essential role in the breakdown of polyphenolic structures into a collection of polyphenolic metabolites of low atomic weight that can be easily absorbed and deliberately be beneficial to health (8). The inter-individual difference in the gut microbiota can prompt contrasts in bioefficacy and bioavailability of polyphenols and their metabolites, in addition to the interindividual variety in the daily admission of polyphenols. Polyphenols are often retained in the gut for more extended periods due to low absorption, where they can have a beneficial effect, especially by influencing the ecology of the intestine (13). During the most recent period, the effect of dietary polyphenols (Figure 1) on the gut ecology and mechanism underlying the impacts on intestinal and extraintestinal diseases is studied (15). In various dietary polyphenols, various researchers have found behaviors such as antioxidant, antidiabetic, anticarcinogenic, neuroprotective, anti-inflammatory, cardioprotective, antimicrobial, antiadipogenic, etc. (16–23).

**Table 1:** Different types of natural polyphenols (7).

**Types**	**Example**		
Flavonoids	Isoflavonoids	Daidzein, ginlycitein Genistein	
	Flavonols	Kaempfer, quercetin Isorhamnetin	
	Flavones	Luteol, ainpigenin Rutin	
	Flavanones	Flavanones, naringen, ninaringin Hesperidin	
	Flavanols	(–)-Epicatechin (+)-Gallocatechin (+)-Catechin	
	Flavononols	Genist, ainstilbin Engeletin	
	Anthocyanidins	Delphinid, pinelargonidin Cyanidin	
Non-flavonoids	Phenolic acids	Hydroxybenzoic acid derivatives	Gallic acid Vanillic acid Syringic acid Ellagic acid
		Hydroxycinnamic acid derivatives	Caffeic acid Ferulic acid Chlorogenic acid p-Coumaric acid
	Lignans	Pinoresinol	
	Stilbenes	Resveratrol	
	Tannins	Hydrolyzable tannins	Ellagitannins Gallotannins
		Condensed tannins	Mono-, di-, trimers 4–6 mers, 7–10 mers, polymers
	Coumarins	6-Methoxymellein6-hydroxymellein	

**Table 2:** Chemical structure of some common dietary polyphenols.

**Dietary polyphenol groups**	**Name**	**Structure**
Phenolic acids	Protocatechuic acid, R = H Vanillic acid, R = OCH3	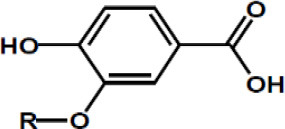
	Gallic acid, R = H Syringic acid, R = OCH3	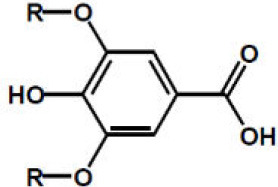
	p-Coumaric acid	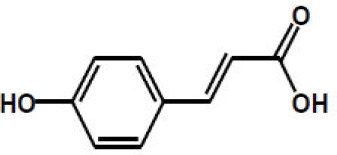
	Caffeic acid, R = H Chlorogenic acid, R = 5-quinoyl Cryptochlorogenic acid, R = 4-quinoyl Neochlorogenic acid, R = 3-quinoyl	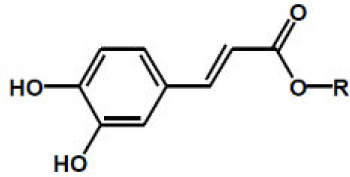
	Ferulic acid	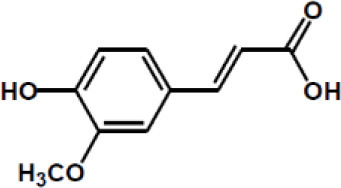
	Sinapic acid	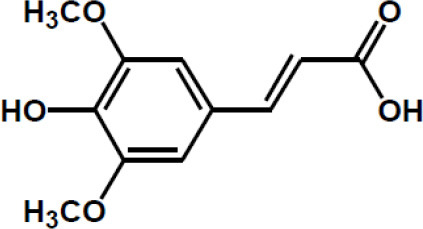
	Rosmarinic acid	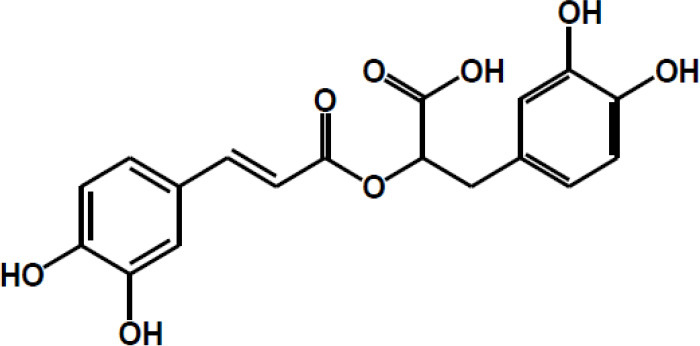
Flavonoid groups and its derivatives	Daidzein: R1 = H; R2 = H; R3 = H Formononetin: R1 = H; R2 = H; R3 = OCH3 Glycitein: R1 = H; R2 = OCH3 Genistein: R1 = OH; R2 = H, R3 = H Biochanin A: R1 = OH; R2 = H; R3 = OCH3	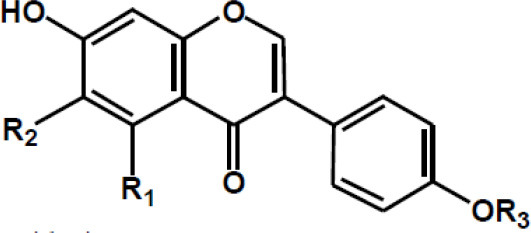
	Dalbergin	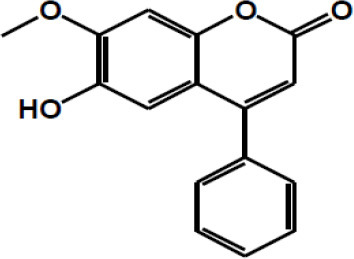
	Phloretin	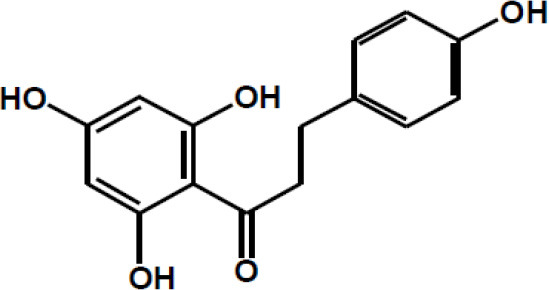
	Xanthohumol	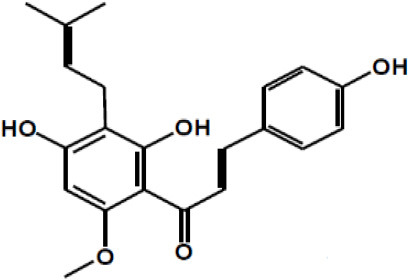
	Apigenin R = H Luteolin R = OH	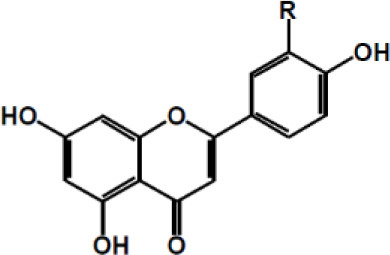
	Tangeretin R = H Nobiletin R = OCH3	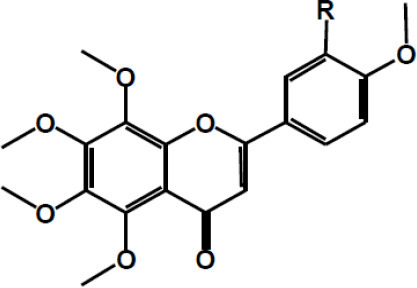
	Naringenin R1 = H, R2 = OH Hesperetin R1 = OH, R2 = OCH3	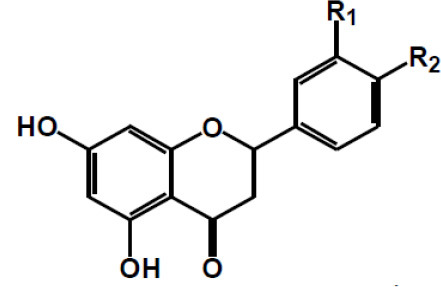
	Kaempferol R1 = H, R2 = H Quercetin R1 = H, R2 = OH Myricetin R1 = OH, R2 = OH Isorhamnetin R1 = OCH3, R2 = H	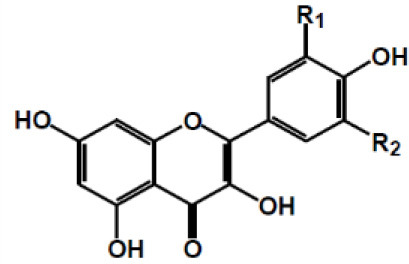
	Taxifolin	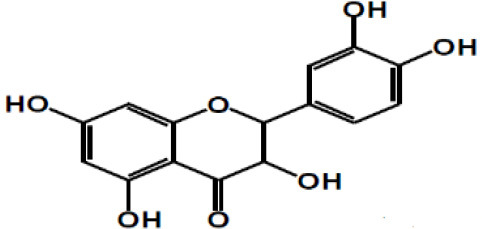
	(+)-Catechin: R1 = R2 = H (+)-Catechin gallate: R1 = gallyl, R2 = H (+)-Gallocatechin: R1 = H, R2 = OH (+)-Gallocatechin gallate: R1 = gallyl, R2 = OH	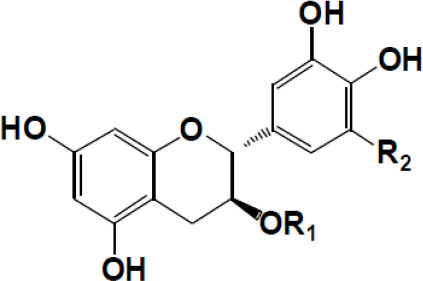
	(–)-Epicatechin: R1 = R2 = H (–)-Epicatechin gallate: R1 = gallyl, R2 = H (–)-Epigallocatechin: R1 = H, R2 = OH (–)-Epiallocatechin gallate: R1 = gallyl, R2 = OH;	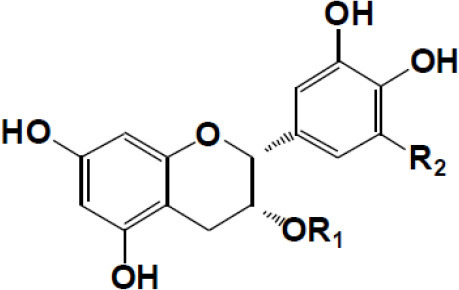
	Procyanidins: *n >* 0 Oligomeric procyanidins: *n =* 0–7	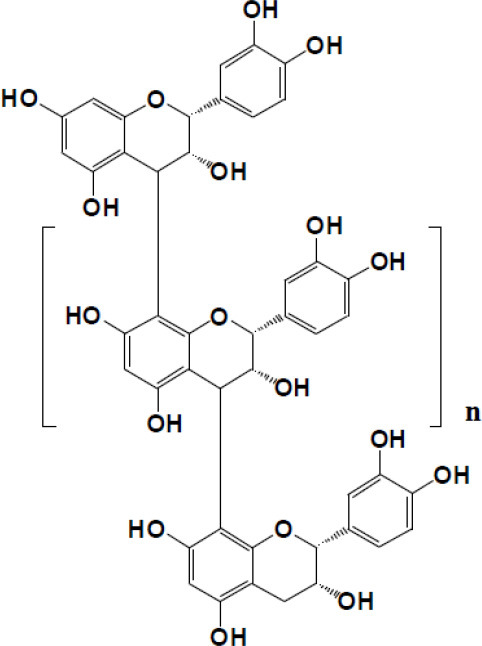
	Theaflavin	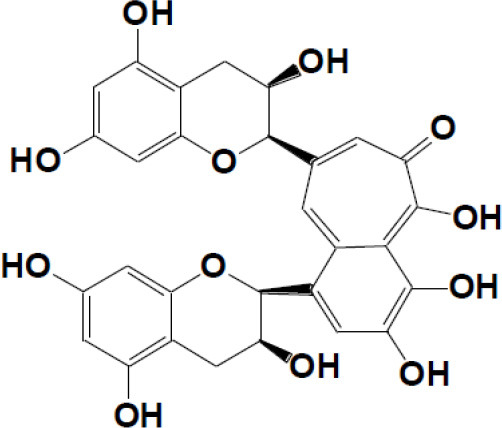
	Anthocyanidins Cyanidin R1 = –OH; R2 = –H Delphinidin R1 = –OH; R2 = –OH Pelargonidin R1 = –H R2 = –H Malvidin R1 = –OCH3 R2 = –OCH3 Peonidin R1 = –OCH3 R2 = –H Petunidin R1 = –OH R2 = –OCH3	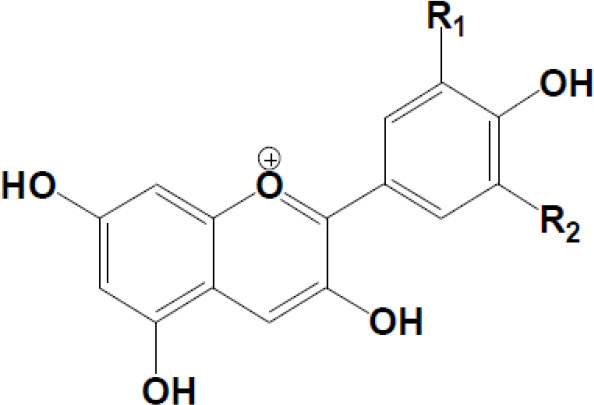
Polyphenolic Amides	Avenanthramide A: R = H Avenanthramide B: R = OCH3 Avenanthramide C: R = OH	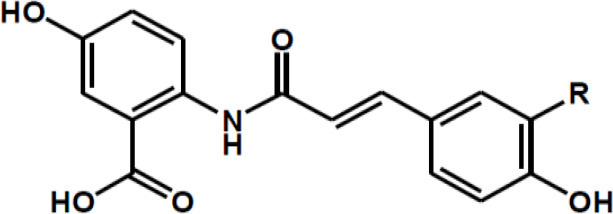
	Capsaicin	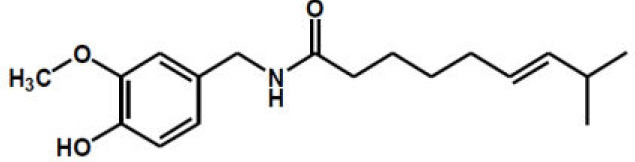
	Dihydrocapsaicin	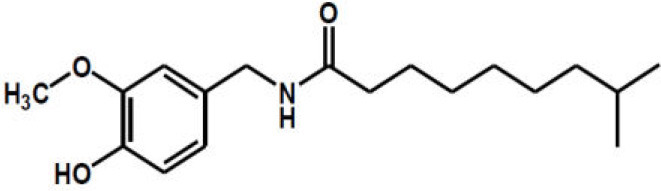
Other major polyphenols	Resveratrol	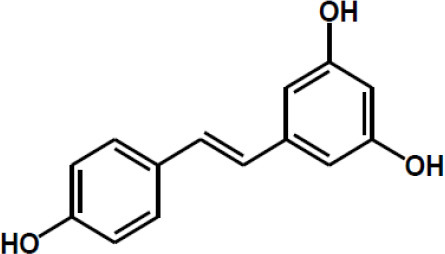
	Curcumin	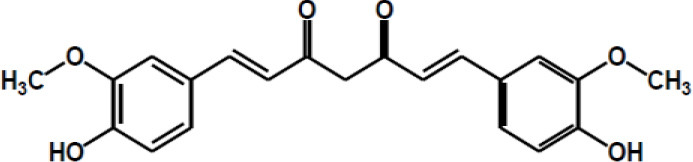
	Gingerol	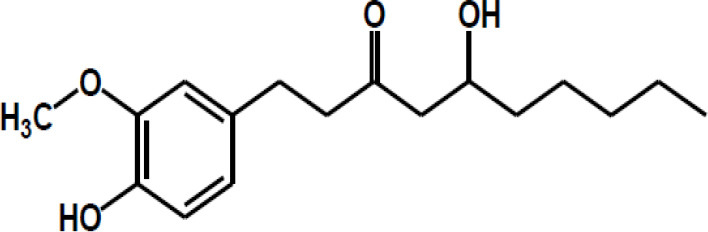
	Ellagic acid	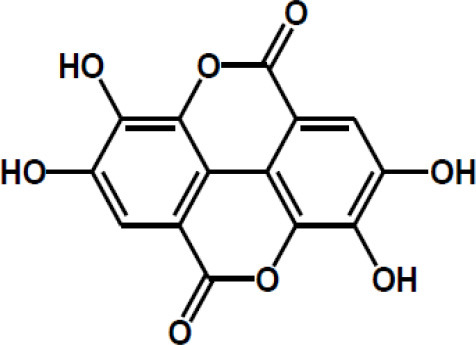
	Matairesinol	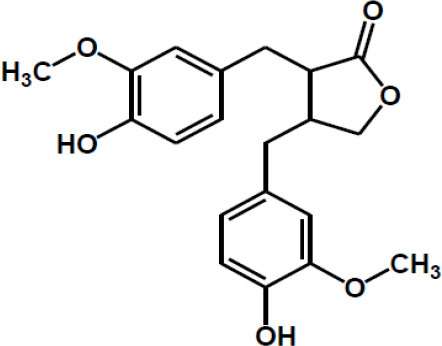
	Valoneic acid dilactone	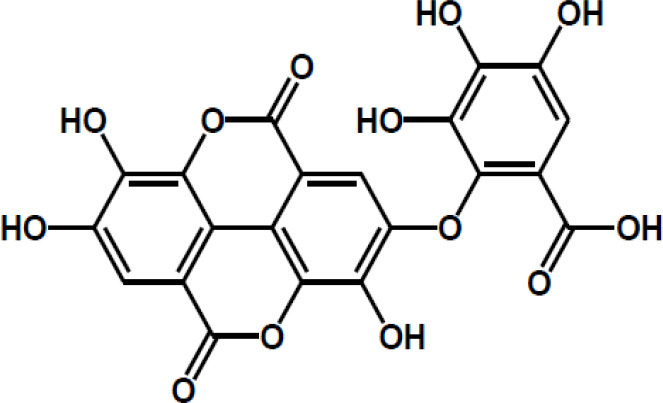
	Secoisolariciresinol	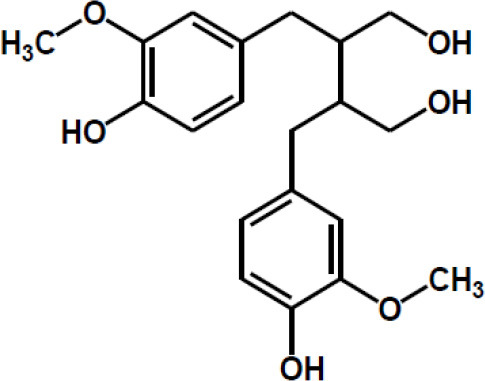

### Polyphenols and Their Biotransformation in Gut

**Figure 2:**
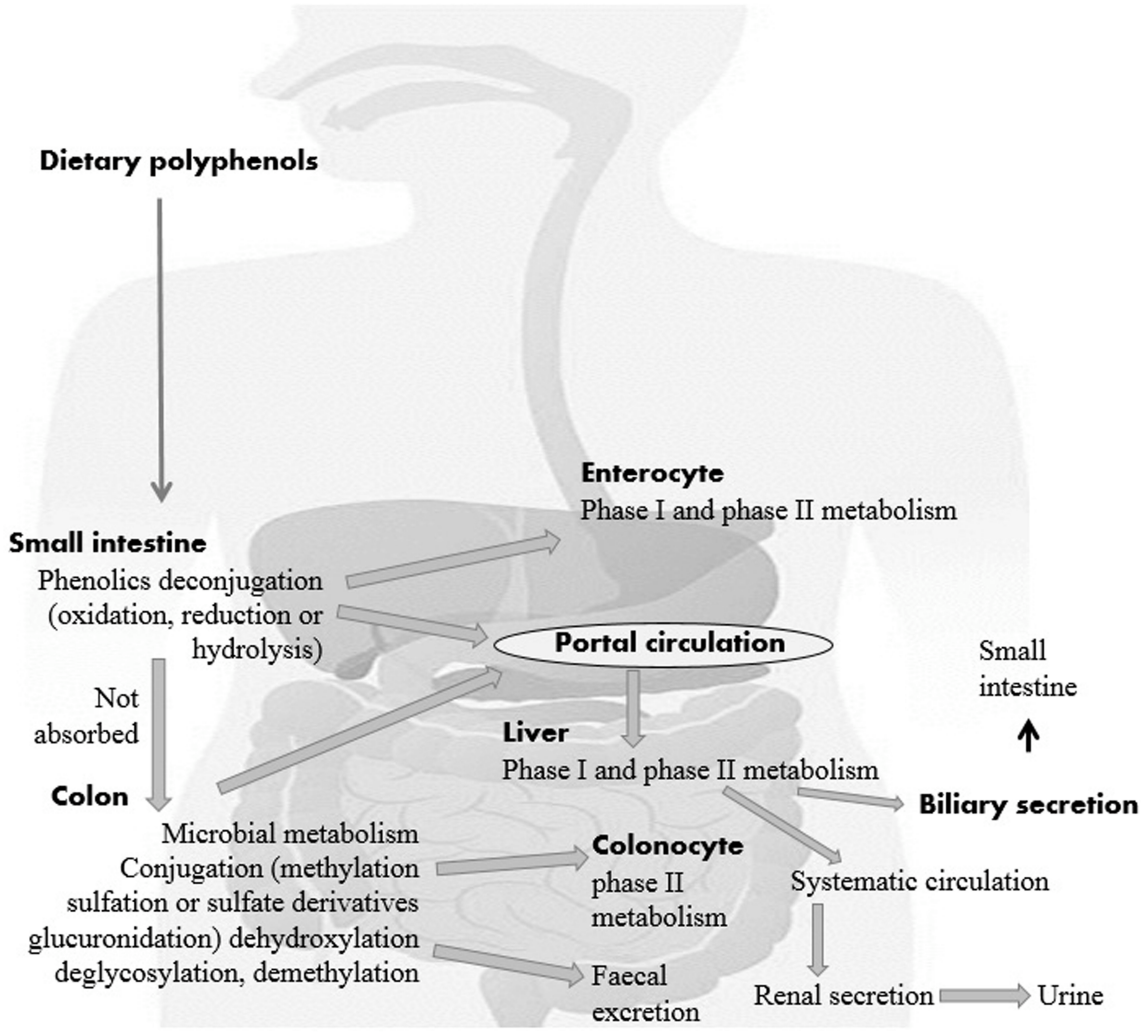
Dietary polyphenols and its digestion.

For each gram of gut material, the human gut harbors an extraordinarily perplexing microbial ecosystem in groupings of 1,012 microorganisms. Every person's gut microbiota composition is unique and is influenced by a legacy acquired at the host's birth, physiological status and genotype, diet, and lifestyle (24, 25). A minor amount of dietary polyphenols (5–10% of total intake) can be easily absorbed using deconjugation reactions such as deglycosylation in the small intestine (26). These polyphenolics may be exposed to large Phase I (oxidation, reduction, hydrolysis, etc.) and Phase II (conjugation) biotransformations in enterocytes after absorption into the small intestine (Figure 2). Then a chain of water-soluble metabolites (methyl, glucuronide, derivatives of sulfate, etc.) rapidly release into the systemic circulation for advanced organ distribution.

Colonic microorganisms are identified to act enzymatically on excess unabsorbed polyphenols in the large intestine, successively creating metabolites of different physiological significance (27). The processing of lactones, phenolic acids, and aromatics with different side chain lengths and hydroxylation, depending on the precursor structures (phenylvaleric acids, phenylacetic acids, phenylvalerolactones, hippuric acids, phenylpropionic acids, benzoic acids, etc.) take place consecutively (28, 29). Besides, in the most recent decade, researchers also studied the transformation of non-flavonoid polymeric molecules called ellagitannins (or hydrolyzable tannins) (30, 31). Tannin structures are exposed to hydrolysis in the intestinal lumen, delivering free ellagic acid, after using ellagitannin-rich foods, such as strawberries and raspberries, pecans, pomegranates, and oaked wines. Once in the large intestine, human colonic microflora metabolizes ellagic acid to produce a series of derivatives known as urolithins, represented by a standard nucleus of 6H-dibenzo[b,d]pyran-6-one and a decreasing number of phenolic hydroxyl groups (urolithin D → C → A → B).

Colonic bioconversion of polyphenols is mostly portrayed for flavonoids. It is a profound factor due to three principal reasons: (a) Variability has been noted in bioconversion of explicit flavonoids (24, 25, 31), and this variability can be attributed to the individual colonic microbiota and has prompted the recognition of low to high flavonoid converters (32, 33); (b) Little contrasts in substitution pattern of flavonoids can initiate significant changes in colonic bioconversion (32, 34); (c) Dietary context of ingested polyphenols that can regulate polyphenol–microbiota (35) interaction.

The first part of our digestive system is the mouth. When we eat polyphenols, the gullet (esophagus) passes into the stomach, and then polyphenol is transferred into the small intestine, and the intestine—from the moment it is first ingested until it is consumed by the body or passed off as feces. Although certain foods and liquids are absorbed through the lining of the stomach, the rest are immersed in the small intestine, like polyphenols. Saliva helps to lubricate food and includes enzymes that begin to digest our food chemically, including food-containing polyphenols. In the colon, a large (~4^13^) number of microorganisms are present, where our bodies consist of around the same number of cells (~3^13^). Most animals are living harmlessly; that is, they are commensals. Some are useful because they synthesize vitamins and digest polysaccharides that we do not have enzymes for (providing an estimated 10% of the calories we acquire from our food). In the small intestine, where most nutrients present in the food are ingested, digestion begins. The lumen surface of the small intestinal folds called the villi, lined by simple columnar epithelial cells called enterocytes, where every enterocyte on the cellular apical surface, have shorter microvilli (cytoplasmic membrane extensions) that increase the surface area to allow more nutrient absorption to occur.

The small intestine climate is less harsh, and microbial communities like Lactobacilli, Diphtheroid, fungus Candida, etc., may be found in the small intestine. On the other hand, a diverse and abundant microbiota is found in the large intestine essential for normal function. These microbes include Bacteriodetes (the Bacteroides and Prevotella genera, in particular) and Firmicutes. These microbes support digestion and contribute to the processing of the feces, digestive tract waste, and flatus, the gas generated by the microbial fermentation of undigested food.

### Effects of Dietary Polyphenols on Intonation of Gut Ecology and Cellular Environment

The colonic microbiota could shift polyphenols to bioactive mixes that can affect the intestinal ecology and impact on host health. *In vitro* animal model and human studies suggest that particular dosages of experimentally selected polyphenols can alter the microbial composition of the intestine, and while some bacterial groups may be limited, others can thrive in the biome niche accessible. Phenolic blends change the gut microbiota and consequently alter the balance of bacteroids (36–38). Tzounis et al. (39) studied an *in vitro* model and proposed that monomers of flavan-3-ol such as (+)catechin and (−)epicatechin might be able to influence the enormous intestinal bacterial populace even in the presence of various supplements, such as starches and proteins. Yamakoshi et al. have studied that an extract rich in proanthocyanidin from grape seeds given to an adult for about 14 days has been able to increase the number of bifidobacteria significantly (40). Recent research indicates that monomeric sources rich in flavan-3-ols and flavan-3-ol, such as chocolate, green tea, and blackcurrant or grape seed extracts, can modulate the intestinal microbiota (39, 41–43). A human intercession analysis found that the use of red wine polyphenols substantially increased the number of bacteria groups, including Enterococcus, Prevotella, Bifidobacterium, Bacteroides, Bacteroides uniformis, Eggerthella, etc. (44). After the use of a wild blueberry drink, Vendrame et al. observed a substantial increase in the amount of Bifidobacterium, recommending a significant role for intestinal microbiota in this case (45). The role of grape seed flavan-3-ols in the production of intestinal bacterial groups using *in vitro* experiments were analyzed by Cueva et al. (46). Although the exact mechanisms need to be clarified, preclinical and clinical evidence indicate that dietary polyphenols have prebiotic properties and antimicrobial activities against pathogenic gut microbiota, which may help with a variety of disorders. Dietary polyphenols, in particular, have been shown to modulate gut microbiota composition and function, interfering with bacterial quorum sensing, membrane permeability, and making bacteria more susceptible to xenobiotics.

### Overview of the Human Gut Microbiota

**Figure 3:**
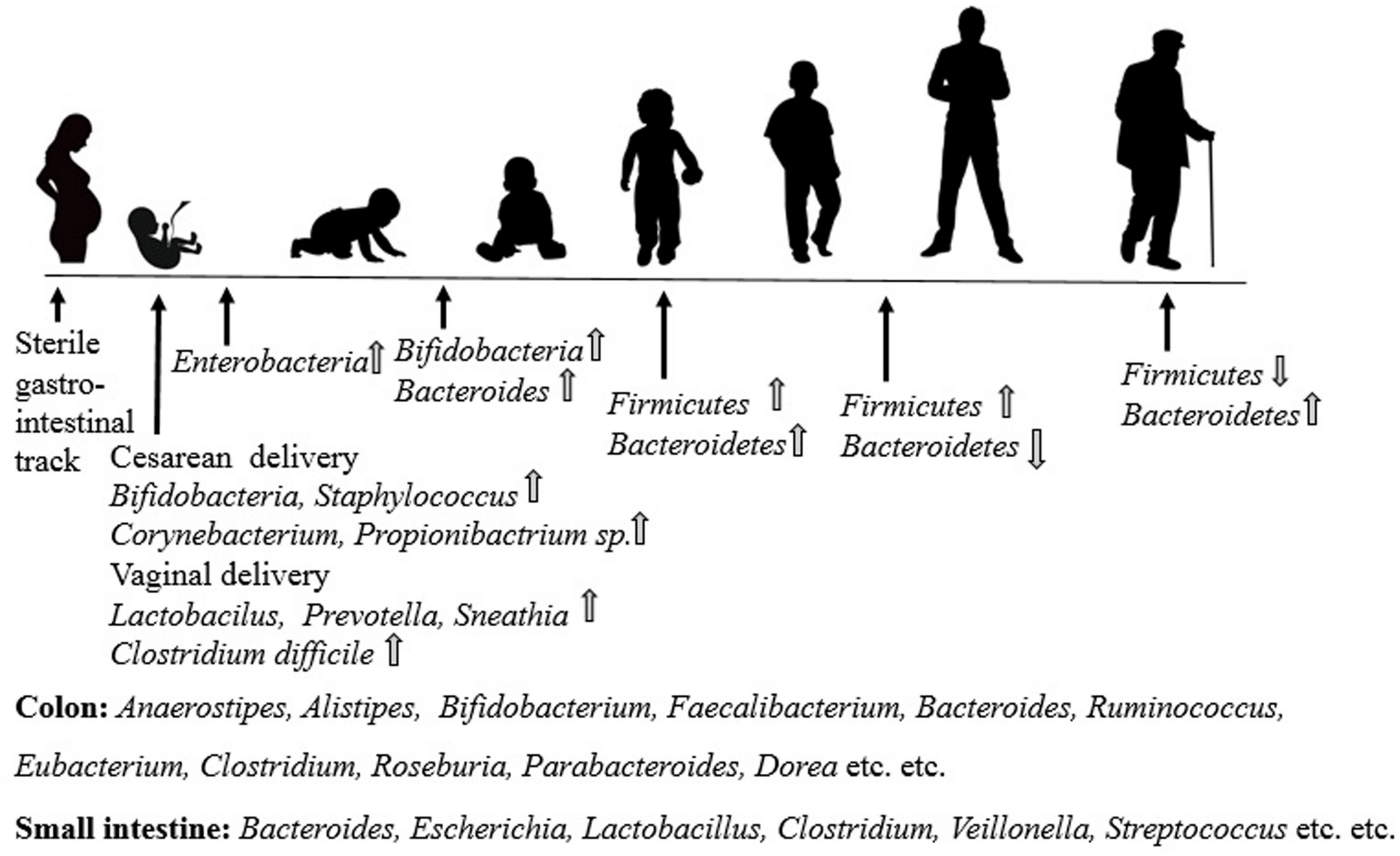
Brief overview of the gut microbiota in different phases of life.

Colonization and early stages of gut microbiota in the infant can be chaotic as the diversity grows over time. Interpersonal variability was found to be higher in newborn children relative to adults because of these complex shifts (47). Neonates establish a gut microbiota that takes into account the high concentration of bifidobacteria in which this dominance prevents pathogenic microorganisms from becoming colonized by competitive exclusion (48). The composition of the gut microbiota (Figure 3) in newborns changes to an adult-like form more than likely after 1 year due to the presentation of foods and other table nourishments, prompting a microbiota dominated by Bacteroidetes and Firmicutes. Finally, by 2.5–4 years old (47, 49), it turns out to be absolutely like the adult microbiota, and the microbiota retain its power until old age after arriving at a developed point. Besides, older individuals have been found to have a different microbial composition compared with young adults, especially *Bacteroides* sp and *Clostridium* sp (50, 51). The role of gut microbiota in people's health and diseases is essential. The sound gut microbiota (7) has been shown to exhibit a particular role in (a) digestion of supplements, (b) xenobiotic and drug metabolism, (c) antimicrobial activity, and (d) immunomodulation.

### Beneficial Effects of Dietary Polyphenols

**Figure 4:**
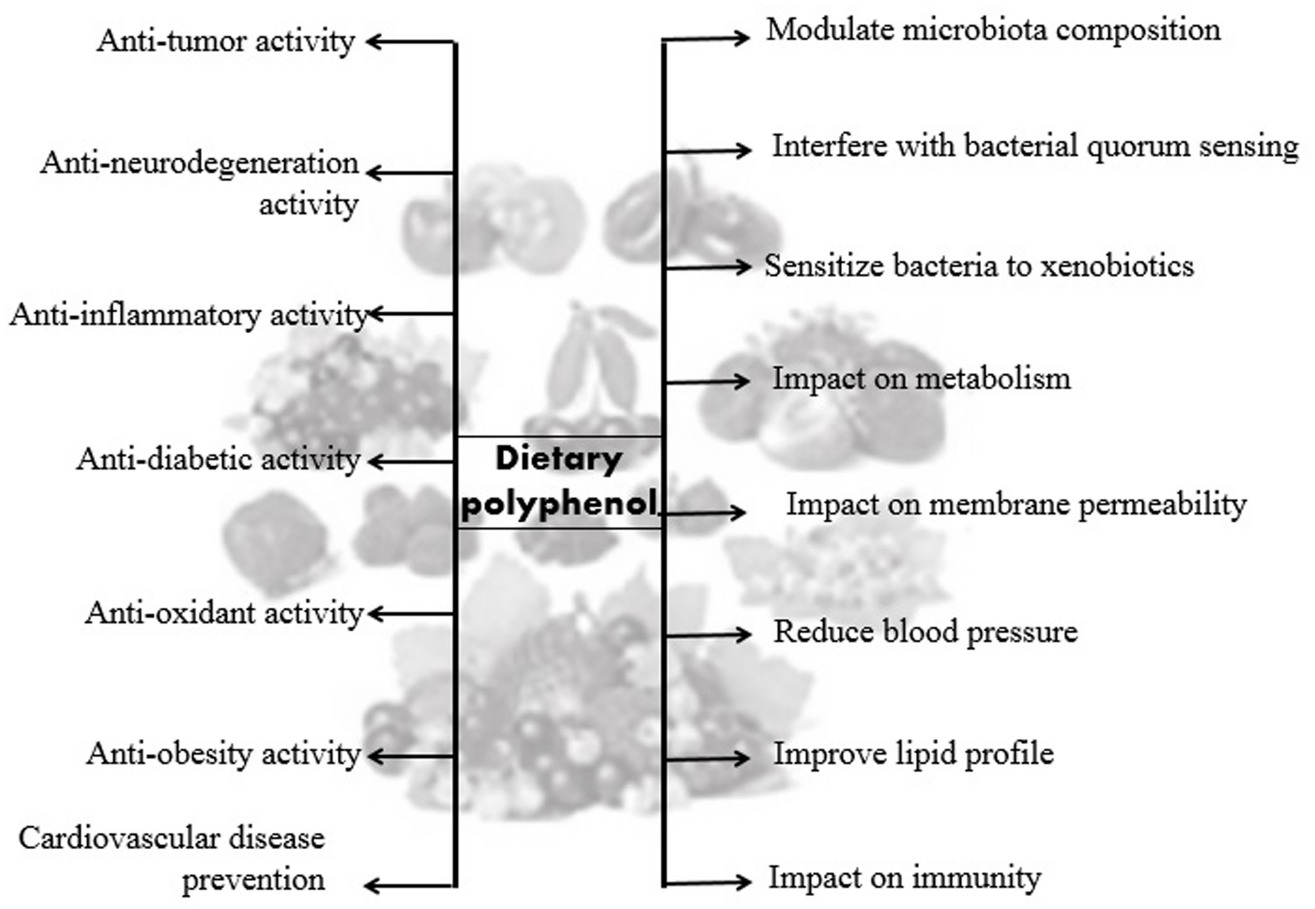
Beneficial effects of dietary polyphenols.

Dietary polyphenols are a large group of bioactive phytochemicals typically found in a wide range of vegetables, fruits, nuts, spices, and drinks, as well as in dry legumes and cereals (52). These are often associated with plant pathogen defenses, and we have a great interest in them because of their medical benefits to humans (Figure 4). They are secondary metabolites and incorporate various molecules with polyphenol structures, with more than 8,000 fundamental variations. As shown by the number of phenolic rings and their side chains or rings, they are split into separate chemical groups (53). Flavonoids have a standard diphenyl propane carbon skeleton in which a linear three-carbon chain interconnects two benzene rings. They can be split into a few subclasses that depend on the state of oxidation of the central pyran ring: flavonols, flavones, anthocyanidins, flavanones, flavonols, and isoflavones. Phenolic acids are classified into benzoic acid derivatives such as protocatechuic acid, gallic acid, and cinnamic acid derivatives, including caffeic, ferulic, and coumaric acids, are critical classes of non-flavonoids. The lignans formed by oxidative dimerization of two phenylpropane units are another essential non-flavonoid group (54, 55). Flavanols are the best known form of flavonoids in foodstuff, with broccoli, apples, tea, blueberries, onions, and red wine being their most lavish sources (56).

Flavanone-rich common foods are mainly citrus fruits such as lemon, grapefruit, and orange fruit (57). Flavanols are present in tea, blackberries, apples, pistachios, and almonds (58). In red fruits and vegetables (e.g., strawberry, red onion, elderberry, pomegranate, cabbage, raspberry), anthocyanins are water-soluble flavonoids present (59, 60). Olive oil, acerola, apricot, apple, nectar, mango, and papaya are examples of sources of flavones (61, 62). Isoflavones are bioactive mixtures mainly found in the legume family, including dried dates, apricot, currants, mango, plums, new coconuts, and sesame seeds (63, 64), although it has been shown that stilbenes are available in grapes, red wine, and berries (65). For instance, red leafy foods, blackberries, strawberries, dark radish, onions, and tea are sources of phenolic acids (57).

The role of polyphenols in counteracting different diseases, such as diabetes, obesity, neurodegenerative diseases, and cardiovascular diseases has been highlighted in numerous studies (65–70). Polyphenols are antioxidant agents also crucial to balance oxidative stress and chronic inflammation (71). Because of this, several studies have concentrated on their beneficial anti-inflammatory (72, 73), pain-relieving (74) and antimicrobial (54, 75), vasodilatory (76), antiallergenic (77), anticarcinogenic (78) effects, etc.

### Bioavailability of Dietary Polyphenols and Impact of Gut Microbiota

It is noteworthy that it is challenging to estimate dietary entry, and a solitary strategy cannot thoroughly evaluate dietary exposure (79), mainly when focusing on micronutrients and bioactive substances, including polyphenols. Polyphenol intake may be affected by a few different variables, for example, dietary propensities, characteristics of the population (e.g., age, gender, and social components), and geological zone (80). The consumption of polyphenols is closely related to bioavailability and bioaccessibility. In this manner, release of polyphenols from foodstuff during gastrointestinal food processing and bioaccessibility would have a major effect on bioavailability that is likely to be accessible for subsequent metabolic pathways through absorption of phenolic mixes.

The gut microbiota impacts the steadiness of dietary polyphenols through multi enzymatic responses, including sulfation, glucuronidation, deglycosylation, C ring cleavage of benzo-γ pyrone system, decarboxylation, dehydroxylation, and hydrogenation. Most of the O-glycosides transformed to aglycones that form the structures of O-glucuronide and O-sulfate. Catabolic transformations, for example, carbon–carbon division of aromatic rings, hydrogenation, decarboxylation, and dehydroxylation of alkene moieties, are completed by gut organisms at that point. For example, quercetin 3-O-glucoside is converted by the intestinal microscopic organisms to phloroglucinol, protocatechuic acid, and 2,4,6-trihydroxybenzoic acid (81–83). Curcumin is catabolized into the structural arrangements of hydrogenated, O-glucuronide, desmethyl, and O-sulfate (84). Polyphenol bioavailability involves dissolution and absorption, transmission to, and disposal of, target tissues, digestion, and excretion. It is influenced by various factors, such as food content variation, food processing, and also other factors such as genetic, microbial, dietary variables, etc. (85, 86). Enterocytes can be easily absorbed by aglycones, flavonols, anthocyanins, etc. Polyphenols are usually glucuronidated or possibly sulfated (stage II digestion) during intestinal absorption, while digestion at stage I (oxidation/reduction) tends to be minor (87). Besides, most native nutrient polyphenols are in the structure of glycosides (flavonols, flavones, flavonones, isoflavones, and anthocyanins), along with less frequent oligomers (proanthocyanidins), which are not absorbable in the intestinal mucosa (88).

### Gut Dysbiosis and Dietary Polyphenols

Metchnikoff originally coined the word “dysbiosis” during the mid-twentieth century to represent the developments in intestinal microbes, proposing a correlation with immune homeostasis and improvement of intestinal disorders. Dysbiosis can be categorized as (89) reduction in the number of symbionts, (90) outlandish pathobiontic development, and (91) loss of diversity. Several components, including age, diet, stress, medications, and xenobiotics, are responsible for impaired gut ecology and/or dysbiosis (89, 92, 93). The growth and evolution of human gut microbes is a case of ecological succession (47, 94). After an underlying stage of massive new colonization, before a steady climax network is formed, they go through progressive composition and function changes. In this case, polyphenols play a significant role. The disintegration of health including impaired salivary function, assimilation, malnutrition (95), and the intake of polyphenol by the diet are further explanations for age-related alterations in the gut ecology.

Diet is a primary modulator of the gut environment, influencing functions and thereby helping to preserve health or facilitate disease conditions (96). Breast milk, for example, contains some oligosaccharides that allow Lactobacillus and Bifidobacterium to multiply, which are predominant in the gut of the newborn child and could contribute to the development of the immune system. There is growing evidence of a relationship between dysbiosis and diseases, including inherited bowel disease (IBD), colorectal carcinoma, obesity, diabetes, etc. (97–100). The pro-inflammatory and pro-oxidative profile produced in the intestinal lumen and nearby layers is a fundamental relationship between dysbiosis and IBD progression (101, 102). In addition, related uremic substances resulting from intestinal microbial digestion of proteins, amino acids, and various metabolites, including mainly protein-bound phenols and indoles, and the effects of digestion of phenylalanine, tyrosine, and degradation of tryptophan, are present (103).

### Polyphenols, Microbiota, and Cancer

**Figure 5:**
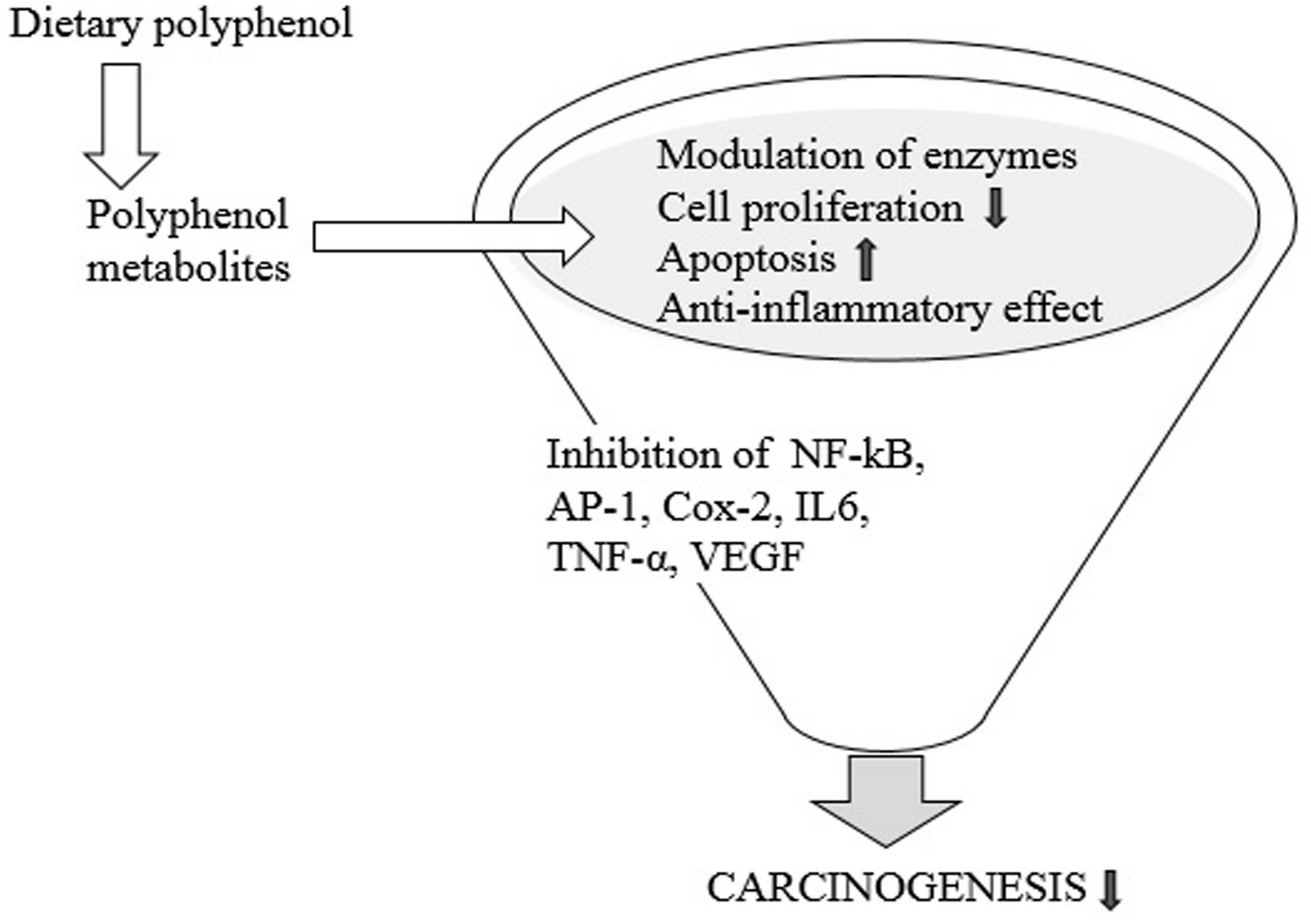
Dietary polyphenol and carcinogenesis.

A few studies have related microbial metabolism of dietary polyphenols to the prevention of malignancy. In patients with and without malignant colorectal development, some studies have reported phylum-level contrasts between the gut microbiota. A few phyla are extended, while others are reduced, but it is not clear exactly how these developments impact the cancer process (104, 105). Studies conducted *in vitro* and gnotobiotic rodent models have shown that gut microbiota may turn plant lignin secoisolariciresinol diglucoside over to enterodiol and enterolactone (31, 106) and protect it in cancer production (Figure 5). Besides, colonization induced by polyphenol significantly decreased tumor number, size, and cell expansion, but increased apoptosis of tumor cells (107). Resveratrol's anti-inflammatory function involves proinflammatory intermediate restriction, eicosanoid union modification, and enzyme obstruction, including COX-2, NF-nB, AP-1, TNF-alpha, IL6, and VEGF (vascular endothelial growth factor) (108). Biological properties, including anticancer and antioxidant function, were accounted for by ellagic acid (109). Some metabolites of intestinal polyphenols such as 3,4-dihydroxyphenylacetic acid and 3-(3,4-dihydroxyphenyl)-propionic acid, quercetin metabolites, chlorogenic acid, caffeic acid, etc., can alter the enzymatic reaction involved in the detoxification of human adenoma cells from LT97. Miene et al. (110) suggested that GSTT2 upregulation and COX-2 downregulation could contribute to the chemopreventive potential of polyphenols in the intestine. Kang et al. reported that by restricting MEK1 and protein kinase such as TOPKK, caffeic acid and coffee directly repressed colon malignant growth metastasis and neoplastic cell transformation in mice (111). It has been found that flavonoids such as quercetin from apples and vegetables have anticancer effects, including cell expansion hindrance and apoptosis induction. One of these nourishments rich in polyphenols is curcumin. Polyphenol-rich foods and the preservation of high microbial diversity have been correlated with the chemopreventive effect of curcumin on the reduction of colonic tumor problems (112).

### Modulation of Gut Ecology by Polyphenols and Impact on Human Gut Health and Immunity

Tzounis and his group revealed that flavonols may have been responsible for decreases in plasma C-receptive protein (CRP) levels, a blood marker of inflammation (41). As of late, Queipo-Ortuño et al. (44) have found that the regular consumption of red wine polyphenols have resulted in significant reductions in blood pressure plasma levels, triglycerides, and high-density lipoprotein cholesterol. Monagas et al. (113) studied phenolic acids such as 3,4-dihydroxyphenylpropionic acid, 3-hydroxyphenylpropionic acid, 3-4-dihydroxyphenylpropionic acid, etc. They have *in vitro* anti-inflammatory activity, reducing the secretion of TNF-α, IL-1β, and IL-6 in peripheral mononuclear blood cells activated by lipopolysaccharides. Larrosa et al. examined the action and anti-inflammatory ability of polyphenols and found that hydrocaffeic, dihydroxyphenylacetic, and hydroferulic acid decreased the development of prostaglandin E2 in IL-1β-mediated colon fibroblast cells (114).

Researchers have indicated that anti-inflammatory activity and intestinal inflammation could be exerted by foods containing essential hydrocaffeic acid precursors such as artichoke, chocolate, apples, and strawberries. Tucsek et al. (115) found that, for example, ferulaldehyde is used to decrease mitogen-enacted protein kinase (MAPK) activity in the final results of polyphenol degradation, thus, repressing NF-βB activity, mitochondrial depolarization, and generation of reactive oxygen species (ROS) (115). Beloborodova et al. (116) have discovered that phenolic acids can be a biomarker of sepsis and that p-hydroxyphenylacetic acid can suppress the production of ROS in neutrophils. Resveratrol and curcumin can change the behavior of B cells as shown by a significant hindrance of lymphokine secretion, antibody production, and expansion (117). Table 3 displays preclinical and human evidence documenting the impact of polyphenols on the gut and related mechanisms.

**Table 3:** Preclinical and human studies with dietary polyphenols on the gut and associated mechanisms (118).

**Experimental model/study**	**Polyphenol**	**Specific condition/concentration**	**Mechanism/effect**	**References**
Bacteria culture	Epicatechin gallate	Incubation in pH 2.0 at 37°C for up to 3.5 h with different concentrations ranging from 2.4 to 8 mg/g	Methicillin-resistant *S. aureus* sensitizes to β-lactam antibiotics	(119)
	Green tea and red wine polyphenols	Concentration ~50–100 mg/culture plate	Inhibits the VacA toxin of *Helicobacter pylori*	(120)
	Crude polyphenols	Different concentrations up to 5 mg/ml	Control of food-borne pathogenic bacteria without inhibitory effect on lactic acid bacteria growth	(121)
Cell culture model	Ellagic acid, genistein, EGCG, resveratrol	Concentration 0.5–1 g/day	Decrease IL-1β-induced IL-8 secretion EGCG reduce the secretion of IL-6 and IL-8 Genistein lowers significantly the levels of IL-6 and MCP-1	(122, 123)
	Green tea, cocoa, and red wine polyphenols	Concentration 0.5 mg/ml	Reduce basolateral IL-6 secretion from Caco-2 monolayers grown on Transwells and challenged with LPS	(124)
	Grape seed, cocoa, sugar cane, oak, mangosteen, and pomegranate polyphenolic extract	Concentration 0.5–4 mg/ml	Reduce IL-1β-induced IL-8 secretion	(125)
	Grifola frondosa water extract	Concentration <100 μg/ml	Inhibits TNF-α and induces MCP-1 and IL-8 in mRNA and protein levels	(126)
	Sardinian wine extracts	Concentration 50–100 μg/ml	Reduce oxysterol-induced IL-6 and IL-8 protein levels	(127)
	Quercetin and pinoresinol	Concentration 100–125 μM working solution	Decrease IL-1β-induced IL-6 and IL-8 levels	(128)
Animal model	Resveratrol	Up to 20 mg/kg body weight	Stimulated fecal cell counts of Lactobacillus and *Bifidobacterium* sp	(114)
			Reduces activities of fecal and host colonic mucosal enzymes, such as α-glucoronidase, nitroreductase, β-galactosidase, mucinase, and α-glucosidase	(129)
			Increases TNF-α and IL-1b level Reduction in IL-10 in colon tissues	(130)
			Increases mucus production in goblet cells in colon mucosa of rat model	(131)
			Decreases IFN-c, TNF-α, IL-6, and IL-1b in serum of mice with acute colitis	(132)
	Quercetin	100 mg/kg	Reduction of body weight Antidiabetic effect	(133, 134)
	Chlorogenic acid	Different concentrations up to 6 mg/ml	Reduce concentrations of TNF-α and IFN-c in jejunum and colon of weaned rats	(135)
	Ellagic acid	Different concentrations up to 0.6 mg/g	Increases mucus production in goblet cells in colon mucosa of rat model of Crohn's disease	(136)
			Reduced the expression of TNF-α and IL-6 in rat colon tissues	(137)
	Coffee and caffeic acid	Different concentrations up to 100 mg/kg	Inhibit colon cancer metastasis and cell transformation in mice by inhibiting TOPK (T-LAK cell-originated protein kinase)	(111)
	Curcumin	120–200 μg/ml	Elevates fecal IgA in rats fed high-fat diet Downregulation of colonic IFN-c and TNF-α levels	(134, 138)
	Proanthocyanidin-rich red wine extract	~250 mg kg^−1^ bw Administered to the rats by orally	Lower levels of Clostridium sp. and higher levels of Bacteroides, Lactobacillus, and *Bifidobacterium* sp	(139)
	Green tea polyphenol	0.15% for 5 weeks	Reduction in the spontaneous release of IFN-c and TNF-α from colon and lamina propria lymphocytes	(140)
	Polyphenols algae	~0.7 g (dry weight)	Decreased counts of Turcibacter and Akkermansia and increase in Alistipes	(141)
	Gallic acid	Different concentrations up to 200 mg/kg	Attenuate mRNA expression levels of TNF-α, IL-1β, IFN-c, IL-6, and IL-17 in colon	(142)
	Red wine polyphenol		Decrease the expression of zinc deficiency Induce TNF-α, cytokine-induced neutrophil Chemoattractant	(143)
	Chinese propolis, Brazilian propolis	100–200 mg/kg/twice daily for 12 weeks	Modulation of gut microflora composition Reduction of *Bacteroides* sp.	(144)
	Piceatannol		Increases in IL-1β, IL-6, and TNF-α in colon	(145)
	Polyphenols (from fungi)		Modulation of gut microflora composition	(118)
	Cocoa containing a total polyphenol		Reduces IgA and IL-6 in Peyer's patches and mesenteric lymph nodes	(146, 147)
	*Prunella vulgaris* honey	~20 μg/ml working concentration	Modulation of gut microflora composition, with increased Bacteroidetes/Firmicutes ratio Restoration of Lactobacillus sp. populations	(148)
	Pomegranate polyphenols	~70 mg/ml working concentration	Reduce TNF-α and IL-1β mRNA, and TNF-α, IL-1β, and IL-6 protein levels in intestinal mucosa	(149)
	Aronia polyphenol, haskap polyphenol, bilberry polyphenol		Elevate the amount of fecal IgA in rats	(150)
Human study	Polyphenols (from spices)		Glucose uptake and control of appetite	(151)
	Dihydroxylated phenolic acid		Shows potent anti-inflammatory properties Reduce the secretion of TNF-α, IL-1β, and IL-6	(113)
	Red wine	150 ml/ day women 300 ml/day men	Regular consumption results in a blood pressure reduction, increased lipid profile, and a decrease of uric acid Increase the proliferation of *Bacteroides* sp.	(152)
	Green tea, fruits, vinegar wine	700–900 ml/day green tea But not excessive	Impact of weight reduction along with alteration in microflora of the gut	(153)
	Cocoa-derived flavanols	Wide range of concentrations depending upon health status	Increase growth and proliferation of *Bifidobacterium* sp. and *Lactobacillus* sp.	(41)
	(+)Catechin and (−)Epicatechin	3 g/day	Inhibition of *Clostridium histolyticum* growth Growth of *Lactobacillus* sp. and *Bifidobacterium* sp. remained unchanged	(39)
	Proanthocyanidin-rich grape extract	100–300 mg/day	Important rise in bifidobacterial numbers	(40)

Resveratrol deacetylates the transcriptional factor STAT3 that cannot generate orphan receptor-gamma t (RORγt) related to retinoic acid, which is a transcriptional factor that is essential for lymphocyte differentiation cycle (154). Another anticipated feature of polyphenol in the gut's adaptive immune response may be its activity on Treg cells, demonstrating a fundamental role in recalling and suppressing autoimmunity for immune tolerance. The secretion of IFNγ, tumor necrosis factor alpha (TNFα), and IL-6 and colonic invasion of CD3^+^ T cells, F4/80^+^ macrophages, and CD177^+^ neutrophils are essentially suppressed by chlorogenic acid employing methods to block the active NF-B signaling pathway (155). The activity of polyphenols involves numerous signaling pathways such as MAPK, ERK1/2, p38, NF-kB, JNK, etc. (156, 157).

### Strategies to Improve Efficiency of Dietary Polyphenols in Gut

Low bioavailability illustrates the biggest drawback of polyphenols, which undermines the medical advantages imaginable. Among various remedial methodologies, multiple procedures have recently been proposed to overcome this challenge. Several methods have been suggested to manipulate physiological changes in the gut, such as pH-delicate delivery system, protein linkers, osmotic-controlled, prodrugs, etc. (158). To boost their digestive stability and intestinal transport, Chung et al. (159) used two separate forms of hydroxyl propyl methylcellulose phthalate (HPMCP), namely, S and L, to cover green tea catechins. Bioavailability was investigated in combination with Caco-2 cells in an *in vitro* gastrointestinal model concerning green tea catechins alone; the intestinal transport rate of L-HPMCP demonstrated an expansion of 3.47 times. In the presence of PLGA in a 2:1 weight ratio, Eudragit S100 (ERS100) was also used to acquire curcumin drug delivery microparticles as anti-inflammatory agents in colitis tissues. Carrier showed the efficacy of curcumin on ulcerative colitis (160). Resveratrol fibroin nanoparticles showed high efficacy in reducing myeloperoxidase activity in repressing TNF, IL-1, IL-6, and IL-12 expression. This technique is, therefore, an attractive framework for the controlled arrival of resveratrol in intestinal inflammation by all accounts (161). Similarly, numerous polyphenols and anthocyanins were absorbed by the microcapsules, which protected them from difficult gastrointestinal conditions. Understanding of the polyphenol components may be studied by controlling microbial arrangement in the gut in collaboration with various probiotic strains. It would enable the enhancement of innovative ways to prevent and treat targeted microbiota diseases and epigenetics and metabolomics discoveries, contributing to tailored drug delivery and nutraceuticals.

## Conclusion Remarks and Future Perspectives

The consumption of polyphenols and their bioavailability decides their health effects. Concentration of plasma of these discrete polyphenolic molecules rarely reaches micromolar levels, considering the high abundance of polyphenols in our diet. Up till now, numerous studies have been performed to know the biotransformation by colonic microflora of polyphenols and to classify the microorganisms responsible. It is clear that dietary polyphenols and their metabolites, by promoting the growth of beneficial bacteria and inhibiting pathogenic bacteria, exerting prebiotic-like effects, contribute to preserving gut health through the regulation of the gut microbial balance. Alternatively, inflection of gut microbiota composition by phenolic compounds has also been assessed to make reciprocal interactions between gut microbiota and phenolic compounds.

In conclusion, it will be crucial to use the recent developments in high-throughput transcriptomic, metagenomic, and proteomic approaches to gain a deeper understanding of the interaction between the gut microbiota and dietary polyphenols to identify the genes and microorganisms involved in polyphenol metabolism and, thus, to elucidate the implications of the relationships between polyphenols. Several factors, including unhealthy dietary patterns, can contribute to the disturbance of the balance of microbiota (dysbiosis) associated with gastrointestinal diseases (including IBD) and extraintestinal metabolic disorders, such as obesity and diabetes. Polyphenols are found in a wide variety of healthy foods, including vegetables, fruits, etc. Proof from preclinical and clinical studies indicate prebiotic effects on polyphenols. The positive effects of polyphenol-rich plants, their extracts, and even individual compounds on gut health, which can be used as an alternative method for preventing or treating various diseases linked to oxidative stress and inflammation, are increasingly being seen and emphasized in the literature. Future studies should also consider their metabolites' behavior, which may influence the therapeutic outcome of health and disease, given that polyphenols may undergo significant changes during digestion and absorption and that the changed forms may have different biological properties and forces.

## Author Contributions

SKR and SM: paper writing, concept, and editing.

## Conflict of Interest

The authors declare that the research was conducted in the absence of any commercial or financial relationships that could be construed as a potential conflict of interest.
